# Leucine Intake and Sarcopenia Indicators of an Elderly Group from the Metropolitan Region, Santiago de Chile, Who Participated in the Program for Complementary Food in Older People (PACAM)

**DOI:** 10.3390/nu16203540

**Published:** 2024-10-18

**Authors:** Edson Bustos-Arriagada, Migdalia Caridad Arazo-Rusindo, Gonzalo Rivera-Andrades, Francisco Pérez-Bravo, Oscar Castillo-Valenzuela, Jorge Barros-Velázquez, María Salomé Mariotti-Celis

**Affiliations:** 1 Faculty of Medicine, Nutrition and Dietetics School, Universidad Finis Terrae, Pedro de Valdivia 1509, Providencia, Santiago 7501015, Chile; edsonbustos@uft.cl (E.B.-A.); ocastillo@uft.cl (O.C.-V.); 2 Department of Analytical Chemistry, Nutrition and Food Science, Food Technology Division, School of Veterinary Sciences, University of Santiago de Compostela, Campus Lugo, 27002 Lugo, Spain; jorge.barros@usc.es; 3 Escuela de Nutrición y Dietética, Facultad de Salud, Universidad Santo Tomás, Avda. Ejército Libertador 146, Santiago 8370003, Chile; marazo@santotomas.cl; 4 Institute of Nutrition and Food Technology (INTA), University of Chile, Santiago 7830489, Chile; grivera.nut@gmail.com (G.R.-A.); fperez@inta.uchile.cl (F.P.-B.); 5 Nutrigenomics Laboratory, Nutrition Department, Faculty of Medicine, University of Chile, Santiago 7830489, Chile

**Keywords:** sarcopenia, leucine intake, elderly, muscle strength, muscle mass, nutritional intervention

## Abstract

Background and objective: The global aging population has led to increased noncommunicable diseases, often linked to poor diet and declining muscle strength and mass. This study assessed leucine intake and sarcopenia indicators among 181 adults aged 60–80 in Santiago, Chile, participating in the Program for Complementary Food in Older People (PACAM), with 80% being women. Methods: Sarcopenia was evaluated through muscle strength and mass using the EWGSOP2 criteria for its identification. Results: 78.45% of participants did not meet the recommended leucine intake of 3 g per day and sarcopenia was identified in 17.13% of them. The skeletal muscle index was 48.07%, and hand grip strength was 26.52%, with men showing significantly higher grip strength (48.60% vs. 28.80%, *p* = 0.00). Inadequate leucine intake was more common in those aged 60–75 (85.9%) than those over 75 (*p* = 0.03). No significant differences in BMI, grip strength, or muscle mass were found between those with adequate and inadequate leucine intake, and PACAM food consumption did not significantly affect these parameters. A sub-analysis showed significant differences in grip strength among powdered dairy drink consumers (35.20% vs. 17.80%, *p* = 0.01). Conclusions: Chilean elderly participating in PACAM present a high prevalence of sarcopenia and leucine deficiency among with no notable benefits from PACAM foods intake. Despite the limitations of the local body composition assessment method, this research addresses a critical public health issue in Chile. Future studies should evaluate physical performance and focus on leucine supplementation to clarify its effects on sarcopenia.

## 1. Introduction

For the first time in world history, the proportion of older people (OP) is the fastest-growing segment of the population [1]. Aging is a natural process in humans that brings about significant changes in physiological processes and nutritional needs among OP. Decreased appetite, metabolism, and physical activity are characteristic of this life stage. Likewise, high oxidative stress and mild chronic inflammation are common processes associated with aging [2].

Sarcopenia, characterized by the progressive decrease in skeletal muscle strength, mass, and function, is an age-related condition with high incidence worldwide [3,4]. The prevention and treatment of sarcopenia should adopt a multifactorial approach, considering lifestyle changes, increased physical activity, and adequate nutrition with high-quality proteins [5,6].

Within the group of proteins, branched-chain amino acids (BCAAs), mainly leucine, have shown an inverse relationship between their plasma concentration and the presence of sarcopenia in OP [7]. Leucine has been observed to have specific positive effects on signaling pathways for muscle protein anabolism through the activation of the mammalian target of rapamycin complex 1 (mTORC-1) [8,9], especially when combined with a diet rich in protein and supplemented with leucine [6,10,11,12]. However, the relationship between dietary leucine intake and sarcopenia in OP has been little studied [13].

The Program for Complementary Food in Older People (PACAM) of the state of Chile aims to maintain or improve the nutritional status and functionality of OP by providing free instant foods fortified with micronutrients. This program was modified in November 2021. Before this modification, it delivered products such as powdered soup (Crema Años Dorados) and powdered dairy drink (Bebida Láctea Años Dorados), which, according to limited studies, had little effect on improving the nutritional status of the beneficiaries and exhibited low sensory acceptability [14,15,16]. Therefore, the objective of our study was to describe and evaluate leucine intake and sarcopenia indicators in a group of OP from the city of Santiago, Chile, who participated in the PACAM prior to its modification. This study assesses daily leucine intake in the normal diets of OP concerning sarcopenia indicators, providing valuable insights into dietary patterns and their implications for sarcopenia management.

## 2. Materials and Methods

### 2.1. Setting and Sampling

This observational cross-sectional study was part of the FONDEF-Project ID17AM0018, where the screening resulted in a total of 181 older people from the universe of the 209 centers of food distribution for elderlies financed by the Chilean government in the Metropolitan Region.

### 2.2. Sarcopenia and Muscle Parameters of the Elderly

Sarcopenia indicators were classified based on two main criteria:

(i) Hand grip strength (HGS):

HGS was assessed using a grip strength dynamometer (Lafayette Hydraulic Hand Dynamometers model J00105, Lafayette Instrument Company, Lafayette, IN, USA) with the dominant hand. The highest recorded value was used. The instrument can measure from 0 to 90 kg, with a minimum unit of measurement of 0.5 kg. Suspected sarcopenia was defined as a dominant HGS of less than 27 kg for men and 15 kg for women.

(ii) Appendicular skeletal muscle mass index (SMI):

The SMI was calculated by dividing skeletal muscle mass (SMM) in kilograms by the square of height in meters (kg/m^2^). 

SMI: skeletal muscle mass index [3] = SMM/height^2^ (kg/m^2^).

The cutoff for height-adjusted SMI from the predictive model was less than 7.45 kg/m^2^ for men and 5.88 kg/m^2^ for women [3]. 

SMM (kg): skeletal muscle mass [3] = 0.107(weight-kg) + 0.251(knee height-cm) + 0.197(calf circumference-cm) + 0.047(−hand grip strength-kg) − 0.034(hip circumference-cm) + 3.417(Sex: 1 if Male, 0 if Female) − 0.020(age-years) − 7.646. 

For the diagnosis of sarcopenia, the updated 2019 consensus of the European Working Group on Sarcopenia in Older People (EWGSOP2) [17] was used. EWGSOP2 recommends using low muscle strength as the primary parameter of sarcopenia (i) considering that muscle strength is the most reliable measure of muscle function. The diagnosis is confirmed by the presence of low muscle quantity or quality (ii).

Anthropometric measurements were taken as follows: body weight and height were measured with barefoot volunteers wearing light clothing, using a balance (SECA 803, Hamburg, Germany, precision 0.1 kg) and a fixed stadiometer (SECA 213, Hamburg, Germany, precision 0.1 cm). The body mass index (BMI) was calculated as weight (kg) divided by height (m^2^), and nutritional classifications were described according to the Chilean Health Ministry (MINSAL, Santiago, Chile) policy (MINSAL, 2008). The waist, hips, and calf circumferences were measured in centimeters and millimeters with a non-extensible metallic measuring tape to the nearest 0.1 cm (Lufkin^®^ Tape Measures, OH, USA). Waist circumference (WC) was determined in the immediately superior point of the iliac crest with non-extensible metallic measuring tape to the nearest 0.1 cm. Hip circumference (HC) was measured at the level of the maximum posterior protrusion of the buttocks. Calf circumference (CC) was measured at the center of the widest point of the participants’ legs.

The anthropometric technique for measuring knee height was performed with the subject seated with the knee and ankle forming a 90-degree angle. A knee height caliper graduated in centimeters and millimeters was then placed vertically, below the heel, and the other end on the femoral condyles, just above the knee.

### 2.3. Assessment of Dietary and Leucine Intake in the Elderly

Dietary and leucine intake were assessed using three 24-h dietary recalls (24-hDR) on three non-consecutive days within one week: two weekdays and one weekend day. Each recall captured the intake from the previous day, ensuring proportional representation of weekend and weekday consumption. Each dietary recall was administered by trained interviewers, provided with standardized neutral probing questions according to the Multiple Pass Method (MPM) [18], and accompanied with the graphic portion’s booklet developed by the Universidad de Chile and MINSAL to improve the precision of the obtained information [19]. To standardize the collected measurements, the interviewers were trained by certified nutritionists who simultaneously worked as supervisors of the fieldwork. The homemade measurements obtained in the 24-hDR were converted to grams (g) and milliliters (mL) [20]. The leucine intake was calculated by means of the average of three 24-hDR using the nutritional software Food Processor II version 7.9 (Esha Research, Beaverton, OR, USA). The total daily leucine intake was expressed as g/day and was compared with the recommended daily dose (RDD) of 3 g/day [21]. 

### 2.4. Statistical Methods

Data processing and analysis were conducted using IBM SPSS Statistics 27.0 software (IBM, Corp., Armonk, NY, USA). Continuous variables were expressed as the median and 25th–75th interquartile ranges, and categorical variables (HGS and SMI) were expressed as percentages. The normality of the data distribution was tested using the Kolmogorov-Smirnov test. Comparisons between groups (gender, age, leucine intake, PACAM food consumption) and gender subgroups were performed using the nonparametric Mann-Whitney U test for two independent samples, and the chi-square test was applied for comparing different groups when variables were categorical.

### 2.5. Ethical Considerations

The protocol of this study was reviewed and approved by the Ethics Committee of the Institute of Food Nutrition and Technology (INTA) of the University of Chile (Project identification code 20/2017; approval date: 13 December 2017) and all participants signed an informed consent form.

## 3. Results

The sample consisted of 181 OP (80% women and 20% men) aged 60 to 80 years from the Metropolitan Region of Santiago, Chile; 78.45% of them showed deficiency in consuming the recommended 3 g of leucine per day for muscle anabolism.

According to sarcopenia indicators derived from measurements of muscle strength and mass, the percentages of sarcopenia components in this group were 48.07% for SMI and 26.52% for HGS (Table 1), with HGS significantly higher in men (Table 2).

**Figure 1:**
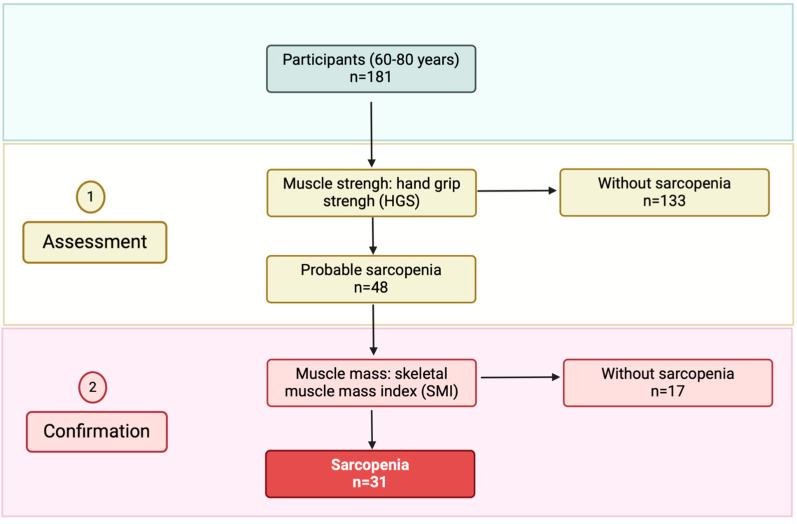
Evaluation of the prevalence of sarcopenia in a group of older people from the Metropolitan Region, Santiago de Chile, who participated in the Program for Complementary Food in Older People (PACAM) according to EWGSOP2 criteria.

Based on the diagnostic criteria for sarcopenia proposed by the EWGSOP2, 31 OP (17 women and 14 men) were diagnosed with sarcopenia according to the indicators of muscle strength and muscle mass (Figure 1). At this stage of evaluation, we can only confirm whether the subject has sarcopenia. However, future research that considers assessment of physical performance is essential for determining the severity of the condition. Additionally, it should also be considered focusing solely on leucine supplementation to better determine its true effects without the presence of confounding factors.

Regarding the effect of age on evaluated parameters (Table 3), 85.9% of OP aged 60–75 years presented an insufficient leucine intake, which was significantly higher compared to those older than 75 years (*p* = 0.03). In contrast, muscle mass variables and sarcopenia indicators showed that older age groups had statistically lower SMM (kg) and SMI (kg/m^2^), with higher percentages for the SMI sarcopenia indicator (35.90% vs. 57.30%, *p* < 0.01).

In terms of compliance with the recommended daily intake (RDD) for leucine and various indicators such as anthropometric measures, muscle strength, muscle mass, and sarcopenia (Table 4), the group with leucine intake ≥ RDD did not show better BMI parameters (kg/m^2^), HGS (kg), SMM (kg), SMI (kg/m^2^), nor lower percentages in sarcopenia indicators (HGS and SMI) compared to those with leucine intake below the RDD.

Regarding the consumption of foods provided by PACAM, 116 OP consumed them. Compliance with the 3 g per day leucine intake was 19.80% for consumers and 24.60% for non-consumers, with no significant differences in BMI, muscle strength tests, or muscle mass between groups (Table 5).

When evaluating the effect of PACAM soup and powdered dairy drink consumption separately (Table 6), only 25 OP consumed the soup, with 29.2% meeting the daily leucine intake recommendation. In contrast, 91 OP consumed the dairy drink, with 19.80% meeting the daily leucine intake recommendation.

However, significant differences were found in the group that only consumed dairy drink, with the HGS sarcopenia indicator showing a higher percentage compared to the non-consumers (35.20% vs. 17.80%, *p* = 0.01).

## 4. Discussion

Our study found high prevalence rates of sarcopenia and inadequate leucine intake among 181 OP aged 60–80 in Santiago, Chile, who were beneficiaries of the PACAM. Specifically, 78.45% did not meet the recommended intake of 3 g of leucine, and sarcopenia was identified in 17.13% of participants. Inadequate leucine intake was more common in those aged 60–75 (85.9%) compared to those over 75 (*p* = 0.03). Men had higher hand grip strength than women (48.60% vs. 28.80%, *p* = 0.00), but no significant differences in body mass index (BMI), hand grip strength, or muscle mass were observed based on leucine intake. These results underscore the importance of dietary protein quality in addressing sarcopenia, particularly as non-communicable chronic diseases linked to aging and poor nutrition become more prevalent. Our focus on leucine intake among PACAM beneficiaries provides critical insights for managing sarcopenia in this vulnerable population. The type of diet, the quality of proteins, and the presence of sarcopenia in OP have gained great relevance today, mainly due to the aging of the global population.

Different reports have been published on the prevalence of sarcopenia in nursing home, hospitalized, and community-dwelling OP [4,22,23]. These studies show that sarcopenia is more prevalent in OP living in nursing homes (51% in men and 31% in women), followed by hospitalized individuals (23% in men and 24% in women), and finally, those who live in community dwellings (11% in men and 9% in women) [4,22,23]. It is important to mention that these studies did not use a single diagnostic criterion, which explains the differences in their results. The prevalence of sarcopenia reported in our sample was 17.12%, being more predominant in men at 37.83% compared to women who presented 11.80%. These results align with those reported by other studies, where the global prevalence of sarcopenia varies between 10% and 27% in people over 60 years of age, being higher in men than in women using the EWGSOP2 criteria [24,25,26,27,28].

On the other hand, many studies relating leucine to the prevention or treatment of sarcopenia in OP have been designed with supplementation models of this amino acid at various doses [29]. Some of these investigations have found a positive effect, where leucine supplementation improved muscle performance, including the skeletal muscle index (SMI), gait speed (GS), and muscle strength [8]. However, little has been studied about the possible effect of dietary leucine intake and its relationship with the prevention of sarcopenia in OP. Ebrahimi-Mousavi S et al. 2022 [13] was the first study that reported the dietary intake of branched-chain amino acids (BCAAs) and its association with sarcopenia in a group of people with a mean age of 66.8 years, showing a prevalence of sarcopenia of 18% using the EWGSOP criteria. The dietary intake of leucine was 5.49 ± 2.25 g/day, which is above the recommended RDD. The SMI was 6.75 ± 0.95 kg/m^2^ vs. 5.95 ± 0.88 kg/m^2^ (*p* < 0.001), and the HGS was 11.30 ± 3.64 PSI vs. 9.85 ± 2.96 PSI (*p* = 0.007) in healthy people vs. subjects with sarcopenia, respectively. When comparing these results with ours, similarities are observed in the prevalence of sarcopenia, but there are significant differences mainly in the daily intake of leucine. In the other study, the average intake meets the RDD of 3 g/day, while our median intake is below this recommendation. In this sense, despite the higher average daily leucine intake in this study, no significant differences were observed in the leucine intake of healthy individuals and those with sarcopenia (5.50 ± 2.20 g/day vs. 5.48 ± 2.48 g/day, *p* = 0.95). Similarly, the prevalence of sarcopenia and its components (HGS, SMI, and GS) was not significantly different between the categories of total and individual BCAAs intake. 

Ángel et al. 2022 [30] reported in their study on trajectories of grip strength and cognition within the Chilean ALEXANDROS cohort that the mean grip strength measured by dynamometry (HGS) was 24.4 kg. In contrast, in our sample, the median grip strength was 20 kg, using the same cut-off points for this sarcopenia indicator. This indicates the lower muscle strength observed in our study and the high percentage of the HGS sarcopenia indicator reported (26.52%).

Regarding the food delivered by PACAM, it was observed that 64.08% of the participants consumed it, with powdered dairy drink being the most consumed product at 78.44%. However, this intake is insufficient to meet the RDD of 3 g of leucine per day, resulting in an 80.20% deficiency of this amino acid. Furthermore, the consumption of PACAM foods and leucine intake had no significant effects on anthropometric indicators, muscle strength, muscle mass, or sarcopenia. Studies carried out in Chile regarding PACAM have shown that 48.10% of the beneficiaries do not collect the food [16]. This group is mainly composed of women, people who live near a health center, those living in urban areas, individuals who do not have routine medical check-ups, and those with health problems, illnesses, or those who have had accidents, including those with permanent or long-term conditions [31]. Masi et al. 2008 [16] reported that the consumption of PACAM foods was 80.00% for the powdered dairy drink and 31.00% for the powdered soup, with a dilution of 52.00% and 28.00%, respectively (like the recommendation of 50.00% for the powdered dairy drink and 25.00% for the powdered soup). However, protein consumption did not present significant differences between the group that consumed the PACAM products and those who did not consume them (63.2 [56.6, 71.3] g/day vs. 49.7 [43.4, 69.5] g/day, *p* > 0.05), respectively. This suggests that factors other than leucine intake could be influencing body composition and sarcopenia outcomes in this population.

In our study, we found a higher prevalence of sarcopenia in men, as well as significant differences in the HGS sarcopenia indicator within this group, particularly among those over 75 years old. Greater deterioration in strength and muscle mass was observed in this age group, despite their higher daily leucine consumption. On the other hand, we did not find any significant relationship between dietary leucine intake and the anthropometric parameters and sarcopenia indicators evaluated. The lack of a clear link between dietary leucine intake and indicators of sarcopenia in this study could be partly explained by the small number of OP with sarcopenia and the low daily leucine intake in the total sample.

The main strengths of our study include the thorough evaluation of daily leucine intake through three 24-hDR (two on weekdays and one on weekends), incorporating information on foods delivered by PACAM. Additionally, we used the current sarcopenia diagnostic criteria proposed by EWGSOP2, making this study one of the few that evaluates leucine intake as an indicator of sarcopenia. However, it also had limitations. It focused on probable rather than confirmed sarcopenia and employed a local method for assessing body composition, which may not be applicable to non-Latin American populations. Additionally, the absence of data on plasma leucine levels, the lack of analysis of participants’ comorbidities, potential biases in the 24-h dietary recall, and the small sample size could influence the findings. Nevertheless, the local algorithm for muscle mass determination is a valuable approach for addressing the needs of the older population in Chile and highlights important public health issues. Future research should further explore physical performance and the effects of leucine supplementation on sarcopenia.

## 5. Conclusions

This study confirms the high prevalence of deterioration in muscle strength and muscle mass in Chilean OP, leading to the diagnosis of sarcopenia. It is also observed that daily intake of dietary leucine in the Metropolitan Region is insufficient compared to international recommendations for OP, regardless of PACAM food consumption. This situation underscores the need for nutritional and public health interventions aimed at improving the quality of products delivered by PACAM and mitigating the negative effects of sarcopenia in older adults. To effectively assess the severity of this age-related decline in skeletal muscle mass and function, future studies must focus on evaluating physical performance in this population. Furthermore, focusing solely on leucine supplementation is crucial for gaining a clearer understanding of its true effects, free from the influence of confounding factors.

## Figures and Tables

**Table 1:** Baseline characteristics of an elderly group (*n* = 181) from the Metropolitan Region, Santiago, Chile.

Evaluated Parameters	Total (*n* = 181)
	Median (25th–75th)
Age (years)	75 (72, 80)
Leucine total daily intake	2.2 (1.6, 2.90)
% < RDD (3 g/day) ^a^	78.5
Anthropometric, muscle strength and physical performance indicators	Median (25th–75th)
BMI (kg/m^2^)	27.3 (24.9, 29.6)
HGS (kg)	20.0 (16.0, 24.0)
SMM (kg) ^b^	14.8 (13.2, 16.5)
SMI (kg/m^2^) ^b^	6.1 (5.6, 6.9)
Sarcopenia indicators	%
HGS (<27 kg for men; <15 kg for women) ^b^	26.5
SMI (<7.45 kg/m^2^ for men; <5.88 kg/m^2^ for women) ^b^	48.1

RDD: recommended daily dose; BMI: body mass index; HGS: hand grip strength; SMM: skeletal muscle mass; SMI: skeletal muscle mass index; ^a^: reference RDD leucine; ^b^: [3].

**Table 2:** Baseline characteristics of Chilean elderly by gender group (*n* = 181).

Evaluated Parameters	Females (*n* = 144)	Males (*n* = 37)	*p*-Value *
	Median (25th–75th)	Median (25th–75th)	
Age (years)	75 (72, 80)	76 (72.0, 79.5)	0.76
Leucine total daily intake	2.2 (1.6, 2.9)	2.2 (1.7, 2.9)	0.62
% < RDD (3 g/day) ^a^	79.2	75.7	0.64 **
Anthropometric, muscle strength, and physical performance indicators	Median (25th–75th)	Median (25th–75th)	*p*-value *
BMI (kg/m^2^)	27.3 (25.1, 30.2)	26.9 (24.2, 28.9)	0.25
HGS (kg)	18.0 (16.0, 21.0)	27.0 (22.0, 31.5)	0.01
SMM (kg) ^b^	13.9 (12.9, 15.3)	19.7 (18.4, 21.7)	0.01
SMI (kg/m^2^) ^b^	6.0 (5.6, 6.4)	7.3 (7.0, 7.7)	0.01
Sarcopenia indicators	%	%	*p*-value **
HGS (<27 kg for men; <15 kg for women) ^b^	28.8	48.6	0.01
SMI (<7.45 kg/m^2^ for men; <5.88 kg/m^2^ for women) ^b^	44.4	62.2	0.05

RDD: recommended daily dose; BMI: body mass index; HGS: hand grip strength; SMM: skeletal muscle mass; SMI: skeletal muscle mass index; ^a^: reference RDD leucine; ^b^: [3]; * *p*-value for U Mann–Whitney between age group; ** *p*-value for chi-square.

**Table 3:** Baseline characteristics of Chilean elderly by age group (*n* = 181).

Evaluated Parameters	60–75 Years (*n* = 78)	>75 Years (*n* = 103)	*p*-Value *
	Median (25th–75th)	Median (25th–75th)	
Leucine total daily intake	1.9 (1.6, 2.4)	2.5 (1.7, 3.1)	0.01
% < RDD (3 g/day) ^a^	85.9	72.8	0.03 **
Anthropometric, muscle strength, and physical performance indicators	Median (25th–75th)	Median (25th–75th)	*p*-value *
BMI (kg/m^2^)	28.33 (25.5, 30.8)	26.8 (24.3, 28.8)	0.02
HGS (kg)	20.0 (16.0, 24.1)	18.0 (16.0, 22.0)	0.07
SMM (kg) ^b^	15.5 (13.8, 16.6)	14.0 (13.0, 16.2)	0.01
SMI (kg/m^2^) ^b^	6.3 (5.9, 6.9)	6.0 (5.6, 6.8)	0.03
Sarcopenia indicators	%	%	*p*-Value **
HGS (<27 kg for men; <15 kg for women) ^b^	20.5	31.1	0.11
SMI (<7.45 kg/m^2^ for men; <5.88 kg/m^2^ for women) ^b^	35.9	57.3	0.01

RDD: recommended daily dose; BMI: body mass index; HGS: hand grip strength; SMM: skeletal muscle mass; SMI: skeletal muscle mass index; ^a^: reference RDD leucine; ^b^: [3]; * *p*-value for U Mann–Whitney between age group; ** *p*-value for chi-square.

**Table 4:** Baseline characteristics of Chilean elderly by Leucine intake (*n* = 181).

Evaluated Parameters	Leucine Intake <RDD ^a^ (*n* = 142)	Leucine Intake ≥RDD ^a^ (*n* = 39)	*p*-Value *
	Median (25th–75th)	Median (25th–75th)	
Age (years)	75.0 (72.0, 79.0)	77.0 (74.0, 81.0)	0.03
Leucine total daily intake	1.9 (1.5, 2.4)	3.6 (3.2, 4.2)	0.01 **
Anthropometric, muscle strength, and physical performance indicators	Median (25th–75th)	Median (25th–75th)	*p*-value *
BMI (kg/m^2^)	27.0 (24.6, 29.8)	27.4 (25.4, 29.2)	0.70
HGS (kg)	20.0 (16.0, 23.3)	18.0 (16.0, 24.0)	0.82
SMM (kg) ^b^	14. (13.22, 16.5)	14.1 (13.3, 17.1)	0.81
SMI (kg/m^2^) ^b^	6.2 (5.6, 6.9)	6.0 (5.6, 6.9)	0.78
Sarcopenia indicators	%	%	*p*-value **
HGS (<27 kg for men; <15 kg for women) ^b^	26.1	28.2	0.79
SMI (<7.45 kg/m^2^ for men; <5.88 kg/m^2^ for women) ^b^	49.3	43.6	0.53

RDD: recommended daily dose; BMI: body mass index; HGS: hand grip strength; SMM: skeletal muscle mass; SMI: skeletal muscle mass index; ^a^: reference RDD leucine; ^b^: [3]; * *p*-value for U Mann–Whitney between age group; ** *p*-value for chi-square.

**Table 5:** Baseline characteristics of Chilean elderly by PACAM foods consumption (*n* = 181).

Evaluated Parameters	Consumes (*n* = 116)	Does Not Consume (*n* = 65)	*p*-Value *
	Median (25th–75th)	Median (25th–75th)	
Age (years)	75 (72, 80)	75 (72, 80)	0.84
Leucine total daily intake	2.2 (1.7, 2.8)	2.1 (1.6, 3.0)	0.84
% ≥ RDD (3 g/day) ^a^	19.8	24.6	0.45 **
Anthropometric, muscle strength, and physical performance indicators	Median (25th–75th)	Median (25th–75th)	*p*-value *
BMI (kg/m^2^)	27.2 (25.1, 29.7)	27.3 (24.4, 29.8)	0.48
HGS (kg)	18.0 (16.0, 22.8)	20.0 (16.5, 24.0)	0.12
SSM (kg)	14.9 (13.2, 16.5)	14.6 (13.2, 16.6)	0.82
SMI (kg/m^2^)	6.2 (5.6, 6.8)	6.1 (5.6, 6.9)	0.71
Sarcopenia indicators	%	%	*p*-value **
HGS (<27 kg for men; <15 kg for women)	30.2	20.0	0.14
SMI (<7.45 kg/m^2^ for men; <5.88 kg/m^2^ for women)	47.4	49.2	0.82

RDD: recommended daily dose; BMI: body mass index; HGS: hand grip strength; SMM: skeletal muscle mass; SMI: skeletal muscle mass index; ^a^: reference RDD leucine; * *p*-value for U Mann–Whitney between age group; ** *p*-value for chi-square.

**Table 6:** Baseline characteristics of Chilean elderly by consumption of PACAM powdered soup and powdered dairy drink.

Evaluated Parameters	Powdered Soup		*p*-Value *	Powdered Dairy Drink		*p*-Value *
	Consumes (*n* = 25)	Does Not Consume (*n* = 156)		Consumes (*n* = 91)	Does Not Consume (*n* = 90)	
	Median (25th–75th)			Median (25th–75th)		
Age (years)	76 (72, 80)	75 (72, 80)	0.80	75.0 (72.0, 80.0)	75.0 (72.0, 79.3)	0.31
Leucine total daily intake	2.4 (1.9, 3.1)	2.2 (1.6, 2.8)	0.09	2.2 (1.8, 2.8)	2.1 (1.6, 3.0)	0.28
% ≥ RDD (3 g/day) ^a^	29.2	20.4	0.33**	19.8	23.3	0.56 **
Anthropometric, muscle strength, and physical performance indicators	Median (25th–75th)		*p*-value *	Median (25th–75th)		*p*-value *
BMI (kg/m^2^)	28.1 (24.7, 29.7)	26.9 (24.9, 29.6)	0.60	27.2 (25.1, 29.6)	27.3 (24.6, 30.1)	0.77
HGS (kg)	18.0 (16.0, 24.3)	20.0 (16.0, 24.0)	0.56	19.0 (15.0, 24.0)	20.0 (16.0, 24.0)	0.24
SMM (kg) ^b^	14.2 (13.1, 16.1)	14.9 (13.2, 16.6)	0.54	14.7 (13.2, 17.1)	14.8 (13.3, 15.9)	0.52
SMI (kg/m^2^) ^b^	6.1 (5.8, 6.9)	6.2 (5.6, 6.9)	0.89	6.2 (5.6, 7.0)	6.1 (5.6, 6.6)	0.41
Sarcopenia indicators	%		*p*-value **	%		*p*-value *
HGS (<27 kg for men; <15 kg for women) ^b^	29.2	26.1	0.75	35.2	17.8	0.01
SMI (<7.45 kg/m^2^ for men; <5.88 kg/m^2^ for women) ^b^	37.5	49.7	0.27	51.6	44.4	0.33

RDD: recommended daily dose; BMI: body mass index; HGS: hand grip strength; SMM: skeletal muscle mass; SMI: skeletal muscle mass index; ^a^: reference RDD leucine; ^b^: [3]; * *p*-value for U Mann–Whitney between age group; ** *p*-value for chi-square.

## Data Availability

All data analyzed in this study are available upon request to the corresponding author. The data are not available publicly due to privacy reasons.

## References

[1] United Nations, Department of Economic and Social Affairs, Population Division (2017). World Population Prospects: The 2017 Revision, Key Findings and Advance Tables.

[2] Barbosa M., Lopes G., Andrade P.B., Valentão P. (2019). Bioprospecting of brown seaweeds for biotechnological applications: Phlorotannin actions in inflammation and allergy network. Trends Food Sci. Technol..

[3] Lera L., Angel B., Sanchez H., Picrin Y., Hormazabal M.J., Quiero A., Albala C. (2015). Validation of cut points of skeletal muscle mass index for identifying sarcopenia in chilean older people. Nutr. Hosp..

[4] Shafiee G., Keshtkar A., Soltani A., Ahadi Z., Larijani B., Heshmat R. (2017). Prevalence of sarcopenia in the world: A systematic review and meta-analysis of general population studies. J. Diabetes Metab. Disord..

[5] Beasley J.M., Shikany J.M., Thomson C.A. (2013). The role of dietary protein intake in the prevention of sarcopenia of aging. Nutr. Clin. Pract..

[6] Beyer I., Mets T., Bautmans I. (2012). Chronic low-grade inflammation and age-related sarcopenia. Curr. Opin. Clin. Nutr. Metab. Care.

[7] Ottestad I., Ulven S.M., Øyri L.K., Sandvei K.S., Gjevestad G.O., Bye A., Sheikh N.A., Biong A.S., Andersen L.F., Holven K.B. (2018). Reduced plasma concentration of branched-chain amino acids in sarcopenic older subjects: A cross-sectional study. Br. J. Nutr..

[8] Ko C.H., Wu S.J., Wang S.T., Chang Y.F., Chang C.S., Kuan T.S., Chuang H.Y., Chang C.M., Chou W., Wu C.H. (2020). Effects of enriched branched-chain amino acid supplementation on sarcopenia. Aging.

[9] Anthony J.C., Yoshizawa F., Anthony T.G., Vary T.C., Jefferson L.S., Kimball S.R. (2000). Leucine stimulates translation initiation in skeletal muscle of postabsorptive rats via a rapamycin-sensitive pathway. J. Nutr..

[10] Chanet A., Verlaan S., Salles J., Giraudet C., Patrac V., Pidou V., Pouyet C., Hafnaoui N., Blot A., Cano N. (2017). Supplementing Breakfast with a Vitamin D and Leucine–Enriched Whey Protein Medical Nutrition Drink Enhances Postprandial Muscle Protein Synthesis and Muscle Mass in Healthy Older Men. J. Nutr..

[11] Hill T.R., Verlaan S., Biesheuvel E., Eastell R., Bauer J.M., Bautmans I., Brandt K., Donini L.M., Maggio M., Mets T. (2019). A Vitamin D, Calcium and Leucine-Enriched Whey Protein Nutritional Supplement Improves Measures of Bone Health in Sarcopenic Non-Malnourished Older Adults: The PROVIDE Study. Calcif. Tissue Int..

[12] Bauer J.M., Verlaan S., Bautmans I., Brandt K., Donini L.M., Maggio M., McMurdo M.E., Mets T., Seal C., Wijers S.L. (2015). Effects of a vitamin D and leucine-enriched whey protein nutritional supplement on measures of sarcopenia in older adults, the PROVIDE study: A randomized, double-blind, placebo-controlled trial. J. Am. Med. Dir. Assoc..

[13] Ebrahimi-Mousavi S., Hashemi R., Bagheri A., Heshmat R., Dorosty-Motlagh A., Esmaillzadeh A. (2022). Association between dietary intake of branched-chain amino acids and sarcopenia and its components: A cross-sectional study. Sci. Rep..

[14] Arazo-Rusindo M.C., Zúñiga R.N., Cortés-Segovia P., Benavides-Valenzuela S., Pérez-Bravo F., Castillo-Valenzuela O., Mariotti-Celis M.S. (2021). Nutritional Status and Serum Levels of Micronutrients in an Elderly Group Who Participate in the Program for Complementary Food in Older People (PACAM) from the Metropolitan Region, Santiago de Chile. Nutrients.

[15] Sanchez H., Albala C., Lera L., Dangour A.D., Uauy R. (2013). Effectiveness of the National Program of Complementary Feeding for older adults in Chile on vitamin B12 status in older adults; secondary outcome analysis from the CENEX Study (ISRCTN48153354). Nutr. J..

[16] Masi C., Atalah E. (2008). Análisis de la aceptabilidad, consumo y aporte nutricional del programa alimentario del adulto mayor. Rev. Médica Chile.

[17] Cruz-Jentoft A.J., Bahat G., Bauer J., Boirie Y., Bruyère O., Cederholm T., Cooper C., Landi F., Rolland Y., Sayer A.A. (2019). Sarcopenia: Revised European consensus on definition and diagnosis. Age Ageing.

[18] Naska A., Lagiou A., Lagiou P. (2017). Dietary assessment methods in epidemiological research: Current state of the art and future prospects. F1000Research.

[19] Universidad de Chile, Facultad de Economía y Negocios, Universidad de Chile, Facultad de Medicina, Ministerio de Salud, Chile (2010). Atlas Fotográfico de Alimentos y Preparaciones Típicas Chilenas: Encuesta Nacional de Consumo Alimentario 2010.

[20] Kim S., Park C.Y. (2023). Validity of Interviewer-Administered 24-h Dietary Recalls in Older Korean Women: A Pilot Study. Nutrients.

[21] Joint WHO/FAO/UNU Expert Consultation (2007). Protein and Amino Acid Requirements in Human Nutrition.

[22] Mayhew A.J., Amog K., Phillips S., Parise G., McNicholas P.D., de Souza R.J., Thabane L., Raina P. (2019). The prevalence of sarcopenia in community-dwelling older adults, an exploration of differences between studies and within definitions: A systematic review and meta-analyses. Age Ageing.

[23] Papadopoulou S.K., Tsintavis P., Potsaki P., Papandreou D. (2020). Differences in the Prevalence of Sarcopenia in Community-Dwelling, Nursing Home and Hospitalized Individuals. A Systematic Review and Meta-Analysis. J. Nutr. Health Aging.

[24] Petermann-Rocha F., Balntzi V., Gray S.R., Lara J., Ho F.K., Pell J.P., Celis-Morales C. (2022). Global prevalence of sarcopenia and severe sarcopenia: A systematic review and meta-analysis. J. Cachexia Sarcopenia Muscle.

[25] Lera L., Albala C., Sánchez H., Angel B., Hormazabal M.J., Márquez C., Arroyo P. (2017). Prevalence of Sarcopenia in Community-Dwelling Chilean Elders According to an Adapted Version of the European Working Group on Sarcopenia in Older People (EWGSOP) Criteria. J. Frailty Aging.

[26] Franzon K., Zethelius B., Cederholm T., Kilander L. (2019). The impact of muscle function, muscle mass and sarcopenia on independent ageing in very old Swedish men. BMC Geriatr..

[27] Kim M., Won C.W. (2019). Prevalence of sarcopenia in community-dwelling older adults using the definition of the European Working Group on Sarcopenia in Older People 2: Findings from the Korean Frailty and Aging Cohort Study. Age Ageing.

[28] Scott D., Johansson J., McMillan L.B., Ebeling P.R., Nordstrom P., Nordstrom A. (2019). Associations of Sarcopenia and Its Components with Bone Structure and Incident Falls in Swedish Older Adults. Calcif. Tissue Int..

[29] Maldonado E.C., Marqués-Jiménez D., Casas-Agustench P., Bach-Faig A. (2022). Efecto de la suplementación con leucina sola, junto con otro nutriente o con ejercicio físico en personas adultas mayores con sarcopenia: Una revisión sistemática. Endocrinol. Diabetes Nutr..

[30] Angel B., Ajnakina O., Albala C., Lera L., Márquez C., Leipold L., Bilovich A., Dobson R., Bendayan R. (2022). Grip Strength Trajectories and Cognition in English and Chilean Older Adults: A Cross-Cohort Study. J. Pers. Med..

[31] Ceroni P., Alvear S., Pino G. (2019). Determinantes de no-participación en el programa de alimentación complementaria de personas mayores, resultados de la CASEN 2015. Rev. Chil. Nutr..

